# Book Reviews

**Published:** 1912-12

**Authors:** 


### BOOK REVIEWS

State Board Examination Questions and Answers, of the U. S. and Canada. 4th edition, 812 pages, $3.00. Wm. Wood & Co., New York.

There is not much opportunity for criticism on a work of this nature unless we hark back to the examining boards. The compilation will appeal especially to undergraduates and candidates for license.

New Aspects of Diabetes, Dr. Carl von Noorden, Vienna. Published by E. B. Treat & Co., New York. 160 pages, $1.50.

This book comprises the lectures at the New York Post-graduate Medical School in Oct., 1912. It has, therefore, the limitation to novel or, at least, recent discoveries and theories and the practicality demanded of the lecturer. So much has been presented in brief form that it is impossible to abstract and, at the same time, preserve the symmetry of the discussion. Neither do we wish to anticipate the careful reading which the book deserves by a random selection of topics. One point, however, we note with pleasure; the ground-word of the glycogenic function of the liver. Some time ago, a student commented on the fact that this function was taught by physiologists and neglected by most writers on diabetes and he wondered if the physiology of the liver dealth with real or imaginary processes.

Year Book of the Pilcher Hospital, April 1, 1911, to March 31, 1912, being the second year of the operation of the hospital. Published by the hospital staff, 145 GatesAve., Brooklyn. 197 pages, illustrated.

The cases are classified as fractures, cancers, benign growths, urologic cases, etc. About half the book consists of appendices, of which those dealing with membranous pericolitis and gastric and doudenal ulcers are especially interesting.

These two books presenting themselves for review at the same time, recall the reports of the Mayos and a few other very elaborate publications of personal or familiar work. Without implying that any of these publications are in any way subject to censure and with due recognition of their high order of merit, it occurs to us that they will very likely establish a precedent for a considerable number of publications involving essentially different standards from those of ordinary periodicals, text books, and institutional reports. This new type of medical publication requires high standards of skill and learning and good judgment and sincerity in its preparation. Even a slight departure from the standards set by the pioneers in this kind of publication might readily lead to a violation at least of the spirit of the ethics of the profession. We suggest, therefore, that it might be wise to anticipate such an event by a very careful consideration of the subject and the statement of principles for future guidance.

---

The Surgical Clinics of John B. Murphy, at Mercy Hospital, Chicago. Vol. 1, No. 5, Oct., 1912 (bi-monthly). Published by the W. B. Saunders Co. $8.00 per year.

The present number contains pages 623-778 and is illustrated with page-plates from photographs and radiograms. It deals with a miscellaneous series of surgical cases.

---

Diseases of the Stomach, Intestines, and Pancreas. By Robert Coleman Kemp, M. D., Professor of Gastro-intertinal Diseases, New York School of Clinical Medicine. Second edition, revised and enlarged. Octavo of 1021 pages, with 388 illustrations. Philadelphia and London. W. B. Saunders Company, 1912. Cloth, $6.50 net; half Morocco, $8.00, net.

The second edition presents many points of improvement over the original, as was prophecied. It should be distinctly understood that Kemp has written no typic stomach book, but has endeavored to set forth all available information with regard to the organs mentioned in the title. Very few authors have viewed this or any other subject from so many aspects. Hence, the reader accustomed to certain kinds of text books which, like the moon, always present the same face to the observer, is likely to be bewildered at first and even to feel indignant that any one knew so many things which he did not know himself. The surgeon, the physiologist, the dietetician, the chemist, and radiologist need this book even more than the clinician, in order to comprehend how small a part each sees of the general subject. There are very few books that represent so much originality and, at the same time, so much careful study of the works of others.

---

A Treatise on Fractures and Dislocations. By Lewis A. Stimson, B. A., M. D., LL. D., Professor of Surgery in Cornell University Medical College, New York. New (7th) edition, thoroughly revised. Octavo, 930 pages, with 459 engravings and 39 plates. Cloth, $5.00, net. Lea & Febiger, Publishers, Philadelphia and New York, 1912.

The author shows a thorough familiarity with anatomy, pathology, and the mechanic principles underlying the successful treatment of displacements whether by fracture or dislocation in the technical sense. There is also evinced, great care in the study of literature and in collating reports of unusual forms of injury. The book belongs to the category of systematic monographs, which attempt one thing and do it thoroughly.

---

The Practical Medicine Series. Under general editorial charge of Dr. Gustavus P. Head and Dr. Charles L. Mix of Chicago. The Year Book Publishers, 180 N. Dearborn St., Chicago. 10 volumes, $10.00 annually.

Vol 5, Series of 1912, Obstetrics, edited by Dr. Joseph B. De Lee and Herbert M. Stowe of Chicago. 229 pages, illustrated, $1.25.

Vol. 6, Series of 1912, General Medicine, edited by Drs. Frank Billings and J. H. Salisbury of Chicago. 350 pages, $1.50.

This review of periodical literature maintains its reputation. It is practical and concise and, since completeness is evidently out of the question, a very fair selection is made.

---

Surgery and Diseases of the Mouth and Jaws. Vilray Papin Blair, A. M., M. D., St. Louis. Published by the C. V. Mosby Co. 637 pages, 384 illustrations, $5.00.

A highly specialized work of this nature properly begins with a general review of the anatomy and pathology and then discusses in sufficient detail to serve as a guide, the various conditions encountered. To the beginner in surgery, almost the same help is given as if the author himself stood behind and directed the operation. Even the surgical expert finds a mass of experience accumulated which is of the greatest value.

---

An Introduction to the Study of Infection and Immunity. Including Serum Therapy, Vaccine Therapy, Chemotherapy and Serum Diagnosis. By Charles E. Simon, M. D., Professor of Clinical Pathology and Experimental Medicine. College of Physicians and Surgeons, Baltimore. Octavo, 301 pages; illustrated. Cloth, $3.50, net. Lea & Febiger, Publishers, Philadelphia and New York, 1912.

We note with regret that the author has followed authority in using the term "immunology." Unlike *appendicitis* and *tuberculosis* this term has not been so long in use that the substitution of the proper word (which we believe would be amynology) would be impracticable. However, when one has made this minor criticism, he has about exhausted the possibilities of the Devil's advocate. The work is systematically arranged and an understanding of a somewhat complicated subject is rendered easy by the clearness of the literary style. The author enters into practical prophylactic and therapeutic problems and presents moot points in a fair manner.

---

MEDICAL RECORD VISITING LIST., Wm. Wood & Co. These memorandum books are old favorites. All contain the customary tables of poisons, denominate numbers, etc. Most of our truthful readers will be well content with the red or black leather folding pocket companion for 30 patients a week with or without dates at $1.25. Others will require those arranged for 60 at $1.50 or for 90 at $2.00. Editions de luxe may also be obtained at $2.50 to $4.00, with extra books to fit inside the cover at 30 to 50 cents. Orders should be sent promptly.

---

THE PRACTITIONER'S VISITING LIST for 1913. An invaluable pocket-sized book containing memoranda and data important for every physician, and ruled blanks for recording every detail of practice. The Weekly, Monthly and 30-Patient Perpetual contain 32 pages of data and 160 pages of classified blanks. The 60-Patient perpetual consists of 256 pages of blanks alone. Each in one wallet-shaped book, bound in flexible leather, with flap and pocket, pencil with rubber, and calendar for two years. Price by mail, postpaid, to any address, $1.25. Thumb-letter index, 25 cents extra. Descriptive circular showing the several styles sent on request. Lea & Febiger, Publishers, Philadelphia and New York.

---

PHYSICIANS' VISITING LIST, P. Blakiston's Son & Co., with dose tables, etc., $1.25.

We are relieved of any possible embarrassment as to showing partiality in reviewing these three visiting lists by the fact that they are all neat and convenient and that they necessarily follow somewhat similar lines. All are well established in use and, doubtless, the selection will be guided mainly with a view to uniformity in the files.

---

A TEXT-BOOK OF PRATICAL THERAPEUTICS. With especial reference to the application of remedial measures to disease and their employment upon a rational basis. By Hobart Amory Hare, M. D., Professor of Therapeutics and Materia Medica in the Jefferson Medical College of Philadelphia. Fourteenth edition, thoroughly revised. Octavo, 984 pages, with 131 engravings, and 8 full-page colored plates. Cloth, $4.00, net. Lea & Febiger, Philadelphia and New York, 1912.

The popularity and excellence of this work are attested by the number of the edition. Part I deals briefly with General Therapeutic Considerations, the old-fashioned classification of drugs according to effect, being placed here. Part II, comprising about half of the volume, discusses drugs alphabetically. Part III of nearly a hundred pages, treats of what used to be termed imponderables, diet and other therapeutic measures than drugs. Part IV devotes nearly three hundred pages to treatment of diseases and symptoms also following an alphatbetic arrangement, and gives the usual tables of dosage, and indexes.

---

MANUAL OF MEDICINE by A. S. Woodwark, M. D., M. R. C. P., London. Published by Henry Frowde and Hodder & Stoughton, Edinburgh, Glasgow and London. (Oxford University Press, American Branch, 35 W. 32d St., N. Y. City) 409 pages, a few illustrations, $3.75.

This is a condensed work intermediate in scope between elaborate treatises on Medical Practice and a quiz compend. Tabular arrangements are used throughout the work, to facilitate reference and enable a quick grasp of the subject. While necessarily much detail, the work is excellent according to its scope

---

A TEXT-BOOK ON THE PRACTICE OF GYNECOLOGY. For Practitioners and Students. By W. Easterly Ashton, M. D., LL. D., Professor of Gyneology in the Medico-Chirurgical College of Philadelphia. Fifth edition, thoroughly revised. Octavo of 1100 pages, with 1050 original line drawings. Philadelphia and London; W. B. Saunders Company, 1912. Cloth, $6.50, net; half Morocco, $8.00, net.

A TEXT-BOOK UPON THE PATHOGENIC BACTERIA AND PROTOZOA. For Students of Medicine and Physicians. By Joseph McFarland, M. D., Professor of Pathology and Bacteriology in the Medico-Chirurgical College, Philadelphia. Seventh edition, thoroughly revised. Octavo of 878 pages, 293 illustrations, a number of them in colors. Philadelphia and London; W. B. Saunders Company, 1912. Cloth, $3.50, net.

PRINCIPLES OF HYGIENE. For Students, Physicians, and Health-Officers. By D. H. Bergey, M. D., First Assistant, Laboratory of Hygiene and Assistant Professor of Bacteriology, University of Pennsylvania. Fourth edition thoroughly revised. Octavo of 529 pages, illustrated. Philadelphia and London; W. B. Saunders Company, 1912. Cloth, $3.00, net.

The above, being well known to the profession, as attested by the exhaustion of several editions, need no extensive review—merely a word of congratulation to authors and publisher.

---

A MANUAL OF AUSCULATION AND PRECUSSION, embracing the Physical Diagnosis of Diseases of the Lungs and Heart, and of Thoracic Aneurysm, and of others parts. By Austin Flint, M. D., LL. D., late Professor of Medicine and of Clinical Medicine in the Bellevue Hospital College, etc., New York. Revised by Haven Emerson, A. M., M D., Associate in Physiology and in Medicine, College of Physicians and Surgeons, Columbia University, N. Y. 12 mo. 361 pages, illustrated. Cloth, $2.00. net Lea & Febiger, Philadelphia and New York, 1912.

This being a revision of a standard text book, needs no recommendation. Austin Flint, the original author of this book, was the son of the founder of the BUFFALO MEDICAL JOURNAL, and was himself the editor from 1857 to 1860. Dr. Emerson has performed the delicate task of modifying another's book, with becoming modesty and good judgment.

A TEXT-BOOK OF OBSTETRICS: INCLUDING RELATED GYNECOLOGIC OPERATIONS. By Barton Cooke Hirst, M. D., Professor of Obstetrics in the University Pennsylvania. Seventh Revised Edition. Octavo of 1013 pages, with 895 illustrations, 53 of them in color. Philadelphia and London; W. B. Saunders Company, 1912. Cloth, $5.00 net; half Morocco, $6.50, net.

We are pleased to bring to the notice of our readers, this excellent work of one of our teachers. It is so well known through earlier editions as to need no elaborate review. Some of the cases of pseudocyesis suggest further study in the light of recent investigations of the hypophysis.

